# Fruits and Farinacea the Proper Food of Man; Being an Attempt to Prove from History, Anatomy, Physiology, and Chemistry, That the Original, Natural, and Best Diet of Man Is Derived from the Vegetable Kingdom

**Published:** 1846-01

**Authors:** 


ART. X.

*Fruits and Farinacea the proper Food of Man; being an attempt to prove from History, Anatomy, Physiology, and Chemistry, that the original, natural, and best Diet of Man is derived from the Vegetable Kingdom.* By JOHN SMITH.—*London,* 1845. 8vo, pp. 418.

THE present age is prolific in popular medical philosophy. Empiricism is not confined to barefaced quackery. There are metaphysicians amongst the mesmerizers and hygienists of a class far superior to the Morisonian. The lay philosophers we allude to have all-grasping minds. Whatever be their peculiar pursuit, they go deep into things. Mesmerism was practised by the early Christians; a fruit and farinaceous diet was the law of paradise. Mesmerism is destined to make manifest the mysterious connexion between matter and spirit; the general adoption of a fruit and farinaceous diet will usher in and characterize the millenium, and enable Great Britain and Ireland to support one hundred and ninety-five millions of inhabitants; that is, if we adopt the estimate and views of John Smith, the Pythagorean of Malton.

Although these extreme opinions are absurd enough, we by no means assert that the inquiries which have led to them are not to be commended. On the contrary, we hail the spirit of medical inquiry amongst laymen as one of the good signs of the times. It is they who will interpenetrate society with medical wisdom; they are the true interpreters and expounders of the esoteric lore of the medical profession, and it is they that will be no trifling instruments for elevating the profession in society.

Mr. Smith is not a mere theoretical writer: he has a claim to be considered a practical philosopher. His attention was directed to the investigation of human diet about ten years ago, after reading an Essay on the manifestations of mind to a small literary society. In that essay he attempted to trace the phenomena of sensation from the lowest to the highest forms of animated being, (one of the characteristic grasps we have just alluded to) and the question occurred to him, "Is man justified in slaughtering animals for his food, seeing that, by a beautifully organized structure, they are rendered exquisitely sensible both of pleasure and pain?" We give the result of this questioning in the author's own words:

"As the subject appeared to me one of great interest, I determined to investigate it as fully as my time, talents, and opportunities would permit; and resolved to adopt practically whatsoever should appear to be the plain dictates of nature. Suffice it to say, that after carefully consulting the writings of Moses, traditionary records, comparative anatomy, physiology, chemistry, general history, and private experience, I arrived at the firm conviction that the flesh of animals is not only unnecessary, but decidedly prejudicial to man's health and well-being. I therefore at once discontinued it as an article of diet, and, notwithstanding the expressed fears and remonstrances of my friends, I persevered; and was soon rewarded with better health and more real enjoyment than I had experienced during many previous years.

"Having derived incalculable advantages from a strict adherence to a fruit and farinaceous diet, and being fully satisfied (after a long and patient investigation of evidence), that it is a food well adapted to *all* constitutions, in *all* climates fit for the residence of man, I can no longer resist the importunity of my friends to make known to the public the result of my inquiries." (Preface, p. xi.)

Thus the public obtained the benefit of John Smith's reading and experience anent a fruit and farinaceous diet; and by this means it fell out that John Smith came under our critical notice. We will hastily follow him through his leading arguments.

It is first argued that fruits and farinacea are the *original* food of man: firstly, because the Mosaic history expressly states that "God said, Behold I have given you every herb bearing seed which is upon the face of all the earth, and every tree in the which is the fruit of a tree yielding seed; to you it shall be for meat." Secondly, because in the works of Greek and Latin writers there are frequent allusions to a period in which man lived in a state of innocence and happiness on the delicious fruits of the earth:

> ———————— "void of care and crime,
> The soft creation slept away their time.
> The teeming earth, yet guiltless of the plough,
> And unprovoked, did fruitful stores allow:
> Content with food which nature freely bred,
> On wildings and on strawberries they fed;
> Cornels and bramble-berries gave the rest,
> And falling acorns furnished out a feast."

This from Ovid, but similar testimony as to the dietetics of the original race of man, and also as to the amazing longevity of these frugivorous people, "is also afforded by Manetho, who wrote the Egyptian History; Berosus, who collected the Chaldean Monuments; Mochus, Hestiæus, Hieronymus the Egyptian, and those who composed the Phœnician History; also by Hecatæus, Hellanicus, Acusilaus, Ephorus, Nicolaus, Diodorus Siculus, Herodotus, Strabo, and Jerome of Egypt."

How long a fruit diet was persisted in, is a matter of question by our author. He thinks, however, that as God had commanded man to be fruitful and multiply and replenish the earth, he would, in obeying this command, be frequently placed in circumstances such that a supply of fruits and farinacea could not be obtained. As man was to have dominion over all animals in all climates, our author thinks it "consistent with all correct views of divine government to expect that he would receive such an organization from the Divine hand, as would render him capable of subsisting on the greatest variety of food—the productions of all climates, with full liberty to use all such as he might be induced by his instincts or reasoning faculties to adopt, as circumstances might require. The flesh of animals, therefore, could not be excepted; for in many climates no other food could be procured." These are Mr. Smith's own words, and yet after expressly stating that man has an organization suitable to the greatest variety of food, he very naively adds, "but we are not thence to infer that the digestive organs of man are the best adapted to an animal or even mixed diet."!

The arguments in the three succeeding chapters as to the original food of man, will be best given in the author's own summary:

"I have now completed my investigations respecting the original diet of man; and have, I trust, satisfactorily proved that the flesh of animals did not contribute to his support. The language of Scripture seems to me particularly clear and decisive on this point, showing that fruit and other vegetables were appropriated to the use of man. His original innocence and moral perfection speak the same language; for the thought of creating pain and misery, by slaughtering an animal in the midst of pleasure and enjoyment, could arise in no breast whereon the image of the Creator was faithfully sealed, except in the case of dire necessity. The testimony of profane antiquity also is in favour of a simple vegetable diet, among the first ages of mankind. The senses of sight, smell, and taste, the instincts expressly designed by the Creator for directing each animal to its appropriate food, loudly proclaim man to have been originally frugivorous; while the absence of fire and other results of discovery would entirely preclude the first human inhabitants of this globe from feasting upon the flesh and blood of slaughtered animals." (p. 44.)

We need scarcely observe that the testimony of profane antiquity is no testimony at all; and that the facts of holy Scripture are not scientific data, and were not written to advance scientific truth. The *philosophy* of the origin of man is as yet involved in impenetrable obscurity, and there seems little probability that science will so advance that the facts recorded by the sacred writer will be satisfactorily explained and elucidated by mere scientific inquiries and speculations. We know certainly that the races of men mentioned in Scripture, and referred to by ancient writers, were Asiatics,—inhabitants of a warm climate,—to whom a fruit and farinaceous diet was alike the most acceptable, the most agreeable, and the best. If then the arguments drawn from these sources be at all valid, (which we however deny altogether,) they are applicable only to the dietetics of inhabitants within the tropics.

The second part is devoted to a series of dissertations, tending to show that fruits and farinacea are the *natural* food of man. These are founded on physiological data. The whole of this part consists of fallacies more or less glaring, which arise out of the indeterminate meaning attached to the term *natural.* This is nowhere defined, but our author and other writers of his class evidently refer to the mythological period termed the golden age, in which men lived in a state of nature. They were (to use a cant phrase,) "robed in innocence and purity;" that is to say, they went about stark naked, like the aborigines of Australia, or Terra del Fuego. They had no houses, but slept beneath "the shade of the forest glade," or perhaps lived like monkeys in the trees from which they derived their sustenance. They had no laws, and of course no law-courts; no wars, no manufactures, no amusements such as civilized man indulges in, no literature, no science. In short, they lived so unlike man as we know man, that they closely resembled the beasts of the field and of the forest. The Indians of California present probably a good example of the "style of living" in the golden age. Their lodges are somewhat like low haycocks, being composed of a framework of sticks thatched with bulrushes; their food consists principally of mussels from the river and of acorns from the forest. Both men and women are to be seen employed shelling, pounding, and baking them into bread; but Commander Wilkes, of the United States' navy, to whom we owe this information, remarks, that the pig-like filthiness with which the process is conducted excites disgust. Man in a state of nature is man in the lowest stage of his development; man in a state of civilization is man in the highest stage of development. From whence then should we take the true type of the genus *homo*? Must we look for it amongst the savages of Australia, or among the nobility and gentry of Great Britain? The folly of such views is amply shown in the following passage, which our author adopts from a kindred writer:

"What is the natural dietetic character of man, according to the real and true evidence of comparative anatomy? In considering this question it is important that we should remember that, whatever may be true concerning the natural dietetic character of man, there is neither now on earth, nor has there been for many centuries, any portion of the human race, so far as we know, which have lived in all respects so perfectly in a state of nature, or in a state to which the constitutional nature of man is most perfectly adapted, as to afford an opportunity to study the true natural history of man, and learn his natural dietetic character from his natural dietetic habits: and therefore, so far as this question is anatomically considered, man must in strict propriety be regarded as an extinct species; because, though man is actually a living species of animal, yet the species, as a whole, have become so artificial in their dietetic habits, that it is impossible to derive from those habits any evidence which can justly be considered unquestionable, in relation to the natural dietetic character of man." (p. 56.)

The conclusion at which our author arrives, is that at which it might have been foretold that he would have arrived, from the premises previously stated. It is sufficiently humiliating:

"Comparative anatomy, therefore, warrants us in concluding that the alimentary organs of the orang are the true type with which to compare those of man, in order to ascertain his true dietetic character. Now, as the orang-outang and most species of monkeys, when in a pure state of nature, and when left free to choose their own food, and to follow their undepraved instincts, are wholly frugivorous—subsisting exclusively on fruits, nuts, and other esculent farinaceous vegetables,—we are perfectly justified by all the laws of correct reasoning in concluding, that the natural food of man is not of that mixed nature which many physiologists would have us believe." (p. 78.)

Professor Owen has, we believe, discovered a striking resemblance between the teeth of the Australian aborigines and the higher simiæ. We know of no race of men so much in "a state of nature" as the blacks of Australia, and we hand over the fact to our author as an additional proof of his views. Indeed, it appears from quotations made by Mr. Smith, that some of the simiæ, like the Australian naturals, cannot resist the temptation of roast beef and other unnatural luxuries.

The authority of celebrated authors is next adduced in support of the phytophagous doctrine, and it is for this reason that our previous remarks were made. Linnæus, Daubenton, who speaks of man in a "state of *pure* nature;" Gassendi, who refers to "the primeval and spotless constitution of our nature;" Sir Everard Home, who thought "while mankind remained in a state of innocence," their only food was of a vegetable origin; are quoted in succession with no other result on our minds, than the conclusion that they all fell in with the old popular mythology of mankind. Even Cuvier speaks of the 'natural' food of man; thus assuming the previous existence of a condition of the human race in which seed and fruit diet was the ordinary diet, and that man was created to use such diet only.

Mr. Smith is well aware of the ordinary objections that can be and are raised against his views, but he seems marvellously ignorant of the utter fallacy lurking under the expressions "natural," and "state of nature." Man differs from all others in being eminently progressive; his natural condition is one of progress; his natural dietetics are founded on a changing power of adaptability to the new circumstances in which his progressive development places him. This adaptability is certainly fully recognized by our author, but its practical results are lost by bending facts to his theory :

"Adapted by nature for feeding upon neither flesh nor herbage, he is (notwithstanding) created with an adaptability to either or both, as climate or circumstances may render necessary; but we are not justified in inferring that he enjoys, by this deviation from nature, that full share of health, pleasure, and longevity, which would be secured by a strict adherence to his more natural diet. If, therefore, we would judge correctly of organs and their functions, we must carefully distinguish between *adaptation* and *adaptability;* and must not hastily conclude that, because an animal can exist and be comparatively well upon a certain kind of diet, it was designed to live on that diet as its best and most natural food. Each animal has been organized upon fixed principles, and each organ has its determinate function and special adaptation; but an all-wise Creator has provided against emergencies, by conferring on each organ—particularly if connected with existence or with organic life—a considerable latitude, by which it can (to a certain extent) vary its functions without destroying its power, or so far impairing the constitution as suddenly to destroy life." (p. 89.)

Here is at once the double assumption that there has been in man a deviation from nature, and that each animal has been organized upon fixed principles of adaptation; whereas, on the one hand we have no scientific knowledge whatever of man's "state of nature," and on the other the tribes, genera, and species of animals pass into each other so frequently, and often so imperceptibly, that adaptability is in fact the law, and appears to determine the generic and specific characteristics. It is by this law that surrounding circumstances act upon the senses and the organs necessary to secure food. At the call of certain common instincts and sensations, the powers of the animal are put into vigorous operation, under circumstances different from those by which itself or its parents had been surrounded. The repeated exercise of these powers develops the organs suitable to their exercise, what was at first strange and displeasing becomes habitual and pleasing, and at last the nature of the animal is adapted to the circumstances. As the circumstances of man with regard to climate, opportunity of obtaining food, clothing, &c., vary greatly, so do his 'nature' and his corporeal form vary; and consequently there is no fixed type or condition invariably applicable to all men.

But even the comparative development of the senses scarcely presents the grounds for Mr. Smith's argument, which he takes up. Having, for example, demonstrated from a consideration of the masticatory organs of the simiæ, that their food is that which is most adapted to the masticatory organs of man, we find him contradicting himself in the consideration of the senses. In man, Mr. Smith observes, we find this sense [the olfactory] placed in a closer relation with fruit than with any other article of diet—a mere assumption, by the by,—and yet in the possession of the maxillary sinus, he is much more allied to the goat and other ruminants than to the simiæ, in whom it is nearly obliterated.

The last argument Mr. Smith introduces in favour of vegetable diet as the natural food of man, is founded on his sensitive and moral feelings. Every manifestation of pain and suffering in a sensitive being, he observes, must at all times awaken the sympathies of the human heart, except in those who are constitutionally obdurate, or whose feelings have been blunted by repeated acts or scenes of cruelty and misery.

"Can we suppose, then, that the Deity would have implanted in the human breast such an aversion to the taking of life, such a horror of shedding blood, and such a heart-sickness on witnessing it; such a hatred of cruelty, and such a sympathy with creatures writhing with pain, if he had intended us to feed upon the flesh of slaughtered animals? Would he not rather have formed us cruel and ferocious, like all carnivorous animals, which seem to derive pleasure from witnessing the sufferings of their victims? Or has the All-wise Creator departed from that harmony of design, so conspicuous in all his works, and rendered necessary for man's support a food, the procuring of which shall do violence to the best and kindliest feelings of his nature; shall be continually weakening and tending to exterminate the attributes of benevolence, mercy, and love; and gradually defacing the image in which God created him? Could he intend that the human race should eat their food with compunction, that every morsel should be purchased with a pang, and every meal empoisoned with remorse? No! Consistency runs through all the works and designs of God!" (p. 114.)

The designs of God have made it requisite that all animals shall destroy each other. Even the herbivorous trample down, crush, and masticate hundreds of insects, while whole nations of men are hunters and fishermen. Indeed, man is a hunting animal; the love of the chase is one of his strongest instincts. So much for the facts on which Mr. Smith grounds his argument. On the other hand, carnivorous animals display warm sympathy for pain and suffering. All persons conversant with dogs know that even the expression of pain on the countenance of those with whom they associate is sufficient to awaken their sympathy, while the cries of suffering will distress the faithful animals exceedingly. In short, the whole argument is merely a display of an effeminancy of sentiment, which would be contemptible were it not well-meaning.

Our Pythagorean having, as he imagines, proved "that vegetables were the original, and are now (as well as in former ages) the natural food of man," he next attempts to demonstrate that they are the best food of man. His arguments are derived from various sources. Firstly, he devotes a chapter to the consideration of the chemistry of dietetics, with a view to show that vegetables contain all the elements and qualities necessary for the complete nutrition of man; that they are easy of digestion, and that "they are superior to animal food or a mixed diet for maintaining all the vital processes; for producing the *mens sana in corpore sano,* in the greatest perfection and for the longest period." The whole chapter shows considerable and praiseworthy research, but the conclusion is lame. It is this:

"Organic chemistry, however, has not yet been brought to such perfection as will enable us to mete out man's food by its laws. We have yet much to learn in this respect; and a short notice of the subject is introduced here only to show that from the vegetable kingdom may be selected, for human food, such articles as will bear a comparison with a mixed diet. so far as our present knowledge will permit us to judge; and that the light already thrown upon the matter by chemistry, is sufficient to prove that fruits, grain, roots, and other esculent vegetables, if used in a natural, unrefined, and unconcentrated state, contain every principle necessary for the nourishment of man." (p. 163)

The next chapter is occupied by proofs that the experience of nations and individuals is in favour of a vegetable diet as the best for man.

Pythagoras heads a list comprising the names of Plutarch, Porphyry, Haller, Ritson, Cheyne, Lambe, Newton, (not Sir Isaac, but one who wrote a work entitled 'Return to Nature,') Shelley, Hufeland, Sir Richard Phillips, sundry Americans, St. Matthew, the three most ancient orders of priests, the Brahmins, Magi, and Druids; and also the Athenian prince Triptolemus, who established the 'Eleusinian mysteries.' The maxims and practice of these persons are set forth at length in the next chapter in proof that the use of fruits and farinacea is conducive to health. Numerous nations in every clime and continent are also referred to in detail, as living almost or altogether on vegetables, and yet being strong, active, and remarkable for their longevity. Our author's style of argument and mode of treating his subject may be gathered from the following extract:

"Examples might be multiplied, from all parts of the world, of people living entirely upon vegetable food, and enjoying perfect health and bodily vigour: but perhaps none are more striking than those we have in close proximity to us. 'The chairmen, porters, and coalheavers, the strongest men in the British dominions, are said to be (the greater part of them) from the lowest rank of people in Ireland, which are generally fed with the potato. No food can afford a more decisive proof of its nourishing quality, or of its being peculiarly suitable to the health of the human constitution.' This remark has been amply confirmed by the recent experiments of Professor Forbes, on the weight, height, and strength of above eight hundred individuals,—his tables clearly showing that the Irish are more developed than the Scotch at a given age, and the English less. The Rev. Howard Malcolm, of Boston, who has travelled extensively in Europe, Asia, and America, says: 'The finest specimens of the human body I ever beheld I saw in Ireland, and they had never tasted animal food.' Many English farmers, who have been in the habit of employing the natives of the emerald isle, bear testimony to the fact, that those who are steady and refrain from spirituous liquors, are indefatigable, and are capable of performing a much greater amount of agricultural labour, on their simple meal of potatoes and butter-milk, than the English labourer, though feeding on abundance of flesh-meat." (p. 222.)

A chapter is headed "Influence of azotized food in the production of certain diseases." The comment is not, however, suitable to the text, as in fact Mr. Smith's arguments are really directed against an *intemperate* use of animal food. Of the baneful effect of such intemperance there can be no doubt whatever. The following paragraph contains so much common sense, that one cannot but feel a little surprise at the frequent aberrations from a sound judgment manifested by the author:

"Without entering further into the nature of disease or its causes, we may show: 1. That too stimulating a diet, or one that is unnatural in quality or quantity, is a very general cause of functional disorder. 2. That an abstemious diet of fruit, grain, and other farinaceous vegetables is, in general, the surest means of restoration to health. Let it however be clearly understood, that improper food is not considered the only means of introducing disease; inattention to exercise, pure air, cleanliness, the cutaneous and other excretions, together with a number of acquired and unnatural habits, may be equally effective in destroying health; and a man who lives temperately upon a mixed diet of animal and vegetable food, and is at the same time regular in other sanitary habits, will enjoy a far greater share of health, and be less liable to epidemic diseases, than the man who adheres to a vegetable diet but neglects all other physiological laws." (p. 239.)

There can be no doubt that the intemperate use of flesh meat induces

(as our author argues) gouty and urinary diseases. It is certain also that there are individuals (as, for example, with a highly arthritic constitution, or who have already been gluttonous) for whom a strictly vegetable diet, may be prescribed with the best result. We think it, however, scarcely warrantable to infer, that a mixed animal diet in moderation induces disease, or to propose that all mankind shall adopt a strictly vegetable diet, because examples of this kind have occasionally occurred. It seems to us much more reasonable to infer, that the exceptions prove the rule. Nor does it follow, that because the flesh of diseased animals is injurious, that flesh must necessarily not be eaten. Fruits and farinaceous vegetables may also be poisonous when diseased. Whole districts sometimes suffer in Germany and other parts of the continent, where rye bread is the ordinary food of the people, from the mixture of ergotted rye. Mr. Smith would, we imagine, think it monstrously unfair to interdict the use of rye bread on this account; and yet this is precisely one of the arguments on which he rejects the use of animal food.

Another reason why animal food should be interdicted is, that it conduces to caries of the teeth. As an example of Mr. Smith's loose mode of argument we quote the following:—

"An intelligent sea-captain, who had visited most parts of our globe, informed Mr. Graham that he found those people who used hot liquids, and hot food, and smoked tobacco and other narcotic substances, always had black and much decayed teeth; but that in the islands of the Pacific, and other parts, where the people seldom or never take anything hot into their mouths, use little or no animal food, and are very simple, plain, and natural in their diet; they had very regular teeth—white, clean, and free from decay. In Mexico the higher classes consume great quantities of animal food, generally eating it three times a day; and they are noted for the early decay of their teeth, and for nervous complaints; whereas the Indians, residing in the same locality, but who live on vegetable produce, are remarkable for their fine white teeth, for their mild expression of countenance, and for their general good health." (p. 273.)

The fact is, that the higher classes in Mexico, of both sexes, *are inveterate smokers*, a reason quite sufficient of itself for the bad state of their teeth. Young Mexican ladies esteem it as much à la mode to smoke cigars as do our English dandies.

A chapter details numerous examples of the beneficial effects of vegetable food on invalids, and will be read with interest. The case of Dr. Lambe, one of the Elects of the College of Physicians, is worthy record:

"In a letter dated April 16, 1825, (?) Dr. Lambe writes as follows:—"From the age of nineteen to thirty-five, I was constantly suffering from the usual symptoms of dyspepsia, which toward the latter period were accompanied by a constant and oppressive pain about the stomach. At the age of thirty-five I had an attack of enteritis, which was severe enough to require two venesections; after this I never went out in the damp of the evening without feeling some tenderness over the abdomen. Under these circumstances, together with a general feebleness of health, I determined to try the effect of substituting distilled water for common water as my drink. The effect of this change was a thorough relief of the dyspeptic pains, and abdominal tenderness. In the ensuing three years, a headache, from which I had occasionally suffered earlier in life, returned so frequently and so severely, as to induce me to take active measures for its relief. I then determined to abstain from animal food, as well as from the use of common water. The intensity of the paroxysms was instantly relieved; yet they recurred in a mitigated form, for at least thirty years. I have been engaged in the active duties of my profession until the middle of last year, which was the eightieth year of my life. Since then, from a partial failure in my sight, I have retired into the country, where, making allowance for my time of life, I enjoy a good share of health." (p. 281.)

We believe Mr. Smith's printer has here substituted 1825 for 1845; as Dr. Lambe's age is certainly nearer 80 than 100. But even if the date were correct, the example of such a Pythagorean centenarian would avail more if we did not occasionally find inveterate smokers and drinkers attain to a longevity not much inferior to this. It is a remarkable circumstance too, developed by the recent sanitary inquiries, that persons attain to an extreme old age, who have been immersed amongst agencies extremely noxious to health; some having been exposed to these all their life; some taking up their residence in the unhealthy localities after middle life.

Mr. Smith next argues that a vegetable diet is protective against epidemics. His facts are of the same one-sided character as those we have previously criticised, or are stated with a carelessness that is scarcely excusable. In opposition to his assertions, we may observe that whole tribes of Indians in Mexico have been swept off by epidemics. The exemption of the negroes of the West Indies from yellow fever is confidently attributed to their use of a vegetable diet! The same remarks apply to the facts adduced in proof that a vegetable diet is conducive to personal symmetry; to the acuteness and perfection of the organs of special sense; and to real sensual pleasures and enjoyment. The case of Casper Hauser, who was fed in a dark dungeon on bread and water, is quoted as an example of the perfection of the senses induced by a vegetable diet; as if the confinement in darkness had no effect in developing the acuteness of vision, &c. observed in that individual. So also in the case of nations remarkable for their physical conformation, no account is taken of the race from which they have sprung, or of their habits. On the other hand, many of the North-American tribes, living almost exclusively on flesh, are remarkable for the fine symmetry of their persons; yet our author takes special care not to allude to them.

The recommendation of a vegetable diet would not be complete, unless it were accompanied by proofs that such a diet was favorable to the government of the passions and propensities, and to the development of man's moral power. These proofs Mr. Smith boldly undertakes to give, and they are like those that have gone before:

"In general," he observes, "those nations and individuals who indulge much in flesh-meat, are more licentious, ferocious, and cruel, than those who subsist on a less stimulating diet; and men noted for barbarity and violence, have an irresistible penchant for animal food, while those who are blessed with milder dispositions, and more benevolent feelings, seem instinctively to adopt a vegetable diet."

This easy assumption of false facts is really amusing. The southern French and Spaniards are by no means remarkable for their gentleness: the latter form probably the most bloodthirsty nation in Europe, yet little flesh do they eat in comparison with the inhabitants of the British isles, more especially the English. In fact, Mr. Smith makes small account of climate, or race, or laws. The Hindoos are gentle, because they abstain from flesh, the carnivora are fierce because they eat flesh, not because they *must* eat flesh, and so on with a hundred examples.

The sixteenth chapter, headed "Diet considered in its relation to population and the moral progress of man," is the most curious in the book. The following extract will show its bearing:

"In the United Kingdom of Great Britain and Ireland there are at present about twenty-eight millions of inhabitants, and about double that number of acres of land in cultivation; consequently, two acres for each individual. Were all living on a full animal diet, the land could only supply food for five millions six hundred thousand inhabitants; on the greatest delicacies of fruits, grain, and roots, one hundred and twelve millions; on grain and other vegetables (where, according to Lord Lauderdale, one acre will support four persons,) two hundred and twenty-four millions; on potatoes and common fruit, five hundred and sixty millions;—without including the extra produce from improved culture. Or let us suppose that, in Great Britain and Ireland, there are (in round numbers) eighty millions of acres, of which sixty millions are arable, or capable of being cultivated. Let half of these be appropriated to the production of the finest fruits, flowers, and timber; and to the support of cattle, sheep, and other animals, for the production of milk, wool, &c.; we shall then have thirty millions of acres for potatoes, wheat, and other grain. Let one half of this remnant be sown with wheat, and the remaining fifteen millions planted with potatoes: then—

| | | |
|---|---|---|
| 15,000,000 of acres of wheat, at 3-qrs. per acre, will feed | 45,000,000 | inhabitants, |
| 15,000,000 of acres of potatoes, at 10 persons per acre, | 150,000,000 | ditto |
| Total . . | 195,000,000 | |

Which is equal to seven times the present population, and more than thirty times the number that the land would support on flesh alone, without taking into consideration the produce of the thirty millions of acres appropriated to fruit and other delicacies. Many useless trees now stand on hedge-rows by the side of common roads; if these were replaced by varieties of apple and pear trees, not only would they be more ornamental, but the owner and the hungry traveller would be supplied with many delightful repasts." (pp. 391-9.)

From this it appears that the euthanasia of the United Kingdom will be consequent on the general adoption of a vegetable diet: that when we have "returned to a state of nature," and live on fruits and farinacea, we shall be a warm snug family of one hundred and ninety-five millions, each living to be as old as the pythagorean centenarian of the College of Physicians. A slight allowance ought have been made for the ground their houses would occupy. If this argument be not sufficiently attractive, Mr. Smith holds out his terrors. Mankind will be suffocated at some future period, unless it take incontinently to fruits and farinacea.

"I may here briefly notice another reason for supposing that man will, in future ages, have recourse to a vegetable diet; though it refers to a period so distant, that it will be deemed worthy of little attention. It is a well ascertained fact, that while plants decompose the carbonic acid contained in the air, and liberate the oxygen, all animals (except the microscopic animalcules) consume the oxygen, and restore the carbonic acid to the atmosphere. Combustion also diminishes the oxygen, and increases the amount of carbonic acid. Now, in proportion as animals multiply, and vegetation decreases, the constitution of the atmosphere must be altered, and rendered less fit for the respiration of man. But it has been shown that, on vegetable food, man requires less oxygen than on animal diet; therefore, by increasing the growth of vegetables for his food, and contracting the numbers of other animals, he preserves the purity of the atmosphere for an increasing human population, and for the continued existence of his species. An *exclusively* animal diet for man, however, is not advocated by any person ; and the calculations are only introduced here for the sake of a comparison." (p. 395.)

Our descendants will certainly want all the fresh air they can get, for if neither wars nor disease interfere with the law of increase of the population for the next two hundred and fifty years, Mr. Smith observes that "eight hundred and ninety-six millions will undoubtedly be the population of Great Britain and Ireland," just about the whole population of the globe!

In conclusion, we have to remark that Mr. Smith believes the millenium is not an improbability, and concludes "that all will then resort to a fruit and farinaceous diet, which is also best adapted to all the wants of the human economy."

"No longer now
He slays the lamb that looks him in the face,
And horribly devours his mangled flesh"

Although we shall still enjoy a quarter of lamb, we wish Mr. Smith all health and happiness on his fare of fruit and farinacea. He writes like a modest, and well-meaning man, and we are sure his book will be useful to wine-bibbers, gluttons, and riotous eaters of flesh. If such wish to know their folly, and to learn wisdom, let them read Mr. Smith's amusing volume.

---

